# Regulation of response to radiotherapy by β-arrestin1 in Non-small cell lung cancer

**DOI:** 10.7150/jca.30012

**Published:** 2019-07-08

**Authors:** Liguang Wang, Kai Wang, Wei Dong, Hongchang Shen, Jiajun Du

**Affiliations:** 1 Institute of Oncology, Shandong Provincial Hospital Affiliated to Shandong University, Shandong Provincial Hospital Affiliated to Shandong First Medical University, Jinan, P.R. China; 2 Department of Oncology, Shandong Provincial Hospital Affiliated to Shandong University, Shandong Provincial Hospital Affiliated to Shandong First Medical University, Jinan, P.R. China; 3 Department of Healthcare Respiratory, Shandong Provincial Hospital Affiliated to Shandong University, Shandong Provincial Hospital Affiliated to Shandong First Medical University, Jinan, P.R. China; 4 Department of Thoracic Surgery, Shandong Provincial Hospital Affiliated to Shandong University, Shandong Provincial Hospital Affiliated to Shandong First Medical University, Jinan, P.R. China

**Keywords:** β-arrestin1, NSCLC, Radiotherapy, DDR, Apoptosis

## Abstract

β-arrestin1 serves as scaffold proteins participating in multiple signaling pathways. However, there were few researches focusing on the impact of β-arrestin1 on DNA damage response (DDR). Non-small cell lung cancer cell (NSCLC) lines were transfected with β-arrestin1 plasmids or siRNA and received radiation treatment. MTT and colony formation assay were performed to assess the proliferation and viability of tumor cells. Flow cytometry was used to evaluate the impact of β-arrestin1 on radiation-induced apoptosis. Western blotting was applied to detect protein expression in apoptosis, DDR, ERK and NF-kB pathways. We used qRT-PCR to test ATR, H2AX, β-arrestin1 mRNA level in cancer tissues compared with para-carcinoma tissues. Co-IP was performed to evaluate the interaction between β-arrestin1 and ATR or H2AX. Comet assay was used to detect DNA damage. β-arrestin1 mRNA level co-related with ATR and H2AX levels in cancer tissues, and β-arrestin1 bound to ATR and H2AX directly or indirectly. Overexpression of β-arrestin1 enhanced the DNA damage response pathway activation and increase DNA damage and apoptosis. Interestingly, suppression of β-arrestin1 inhibited cell proliferation and attenuated ERK and NF-kB pathways activation induced by radiation. Overexpression of β-arrestin1 enhances DDR pathway activation induced by radiation, as well as downstream apoptosis, and depletion of β-arrestin1 inhibits DDR pathway. Meanwhile β-arrestin1 regulates cell proliferation by suppression of ERK and NF-kB pathways. Manipulation of β-arrestin1 status modulates radiosensitivity for NSCLC.

## Introduction

Lung cancer is a major threat to human health all over the world for its high incidence and mortality among cancers 1. In recent years, radiotherapy has made considerable process in techniques for more precise planning, less damage to normal lung tissue and less side effects 2. And radiotherapy is considered to be suitable for the inoperable patients like advanced stage patients, elderly patients, etc 3, 4. Meanwhile, postoperative and preoperative radiotherapy are also important adjuvant treating choice especially for the patients with N2 nodal disease 5. However, radiation resistance, radiation toxicity, long-term metastasis and recurrence restrict its application and attenuate its efficacy 6. Feasible factors relevant to radiation resistance include the amount of tumor stem cells, local hypoxia, potentially lethal damage repair, regulating capacity between survive and apoptosis, accelerated repopulation, intrinsic sensitivity, etc 6-8. Therefore, any measure taken according to the factors above might increase the radiation sensitivity and improve the therapeutic effect. Drugs targeting cell signaling, growth factors and repair of DNA damage may be efficient radiosensitizers 9.

β-arrestins including β-arrestin1and 2 (ARRB1/2) were initially discovered to interact with G protein-coupled receptor (GPCR) to mediate its internalization and desensitization 10, 11. Afterwards, more roles of β-arrestins were found as adaptors and signal transducers in scaffolding and modulating a variety of intracellular signal networks such as p53/Mdm2, MAPK, NF-ĸB, and PI3K/Akt, etc. 12. As such, β-arrestins take part in cell proliferation, apoptosis, angiogenesis, migration and other biological behaviors relevant to tumorigenesis and metastasis 13. Kook reported β-arrestin1 could be cleaved by caspases, then bind to tBID and enhance tBID induced cytochrome C release, thus directly promoting apoptosis 14.

Recently, it was reported that β-arrestin1 was involved in DNA damage induced by a stress response pathway^15^, ^16^, which indicated that β-arrestin1 might take part in DNA damage response. In this study, we aim to identify the role of β-arrestin1in DDR pathway activated by radiotherapy, and explore underline mechanism of regulation effect.

## Materials and Methods

*Participants and Ethic Permission.* All the patients involved gave a signed consent for the study, and the study was approved by the Hospital Ethical Committee of Provincial Hospital afflicted to Shandong University. All methods used in this study were carried out in accordance with the approved guidelines. Fresh frozen tissue samples from 40 patients with primary lung cancer patients who had undergone complete surgical resection were obtained in this study. Patients with NSCLC were included if they met the following criteria: confirmation of NSCLC via a review of pathologic slides by at least two independent pathologists to classify the histologic subtype; no pro-surgical or pro-diagnostic history of anti-neoplastic therapy, including radiotherapy, chemotherapy or targeted therapy; the absence of a second carcinoma, as determined from the clinical history, computed tomography (CT), ultrasonographic examination, and routine laboratory tests. Detail information of enrolled patients was shown in supplementary table 1. Relevant clinical data were acquired from the Bio-Bank of Shandong Provincial Hospital.

For histology and TNM stage, the classification criterion for lung tumors of the World Health Organization and International Association for the Study of Lung Cancer (WHO/IASLC) was applied. For follow-up, patients were evaluated every 3 months by thorax CT and abdomen ultrasonography for the first 2 years after operation and adjuvant treatment, and annual thereafter according to schedule.

*Reagents.* Anti-β-arrestin1, -ATR, -Chk1, -H2AX, -ERK, -PARP, and -NF-kB; anti-phosphorylated ATR (Ser428), Chk1 (Ser345), BRCA1 (Ser1524), ERK (Thr202/Tyr204), γ-H2AX (Ser139), and -NF-kB p65 (Ser536) antibodies were all purchased from Cell Signaling Technology, USA. Anti-GAPDH antibody was purchased from Santa Crus, USA. Calcein AM/Propidium Iodide Double Stain kit was purchased from YEASEN, China.

*Cell culture, transfection, and X-ray irradiation.* All lung cancer cell lines (H1299, A549, H520 and H460) were all purchased from the American Type Culture Collection (ATCC, USA). All cell lines were cultured in RPMI 1640 medium (Hyclone, USA) with 5 % fetal bovine serum (Gibco, USA). All cell lines were cultured in a humidified 37 ºC atmosphere with 5 % CO_2_. For both plasmids (pcDNA and β-arrestin1 (ARRB1+)) and small interfering ribonucleic acid (si-mock and si-β-arrestin1(ARRB1-)) transfection, all cells were seeded into a 6 well cell culture cluster (diameter of each hole was about 35 mm, Corning, USA). When the cell density reached about 50 % to 70 % in the next day, Lipofectamine 2000 transfection reagent (Invitrogen, USA) was applied according to the instructions.

The cells were cultured in serum free medium for 6 hs, and were then exposed to 6 MV X-ray irradiation, produced by a Siemens-Primus linear accelerator Primus 3831 (Siemenscompany, Germany), at a dose rate of 2 Gy/min. The total dose administered was 1, 2, 4 Gy. The cells were then harvested 1, 10 and 72 hs later.

*Quantitative Real-time Polymerase Chain Reaction.* Total RNA was extracted from the carcinoma and para-carcinoma tissues from patients. Then genome DNA was erased from total nucleic acids and mRNA was converted into cDNA using PrimeScript RT reagent Kit with gDNA Eraser (Takara, Japan). Afterwards, each cDNA was amplified with SYBR Premix Ex Taq (Tli RNaseH Plus, Takara, Japan) and analyzed by Roche LightCycler 480II.

18S rRNA sequence Forward 5'-CAGCCACCCGAGATTGAGCA-3' and Reverse 5'-TAGTAGCGACGGGCGGTGTG-3'; ARRB1 sequence Forward 5'-ACAAAGGGACCCGAGTGTTC-3' and Reverse 5'-GCAGGTCAGCGTCACATAGA-3'; ATR sequence Forward 5'-GGAGGAGTTTTGGCCTCCACA-3' and Reverse 5'-CTGCGAGGCACTAGTCAACC-3'; H2AX sequence Forward 5'-AACGACGAGGAGCTCAACAA-3' and Reverse 5'-GCGGGCCCTCTTAGTACTCC-3'.

*MTT assay and Colony formation assay.* MTT assay was used to test cell survival and viability after radiation. Cells were seeded into 96 well culture plates with a density of 5x10^3^ /well. After incubation overnight, the 96 well culture plates received radiation. 48 hs after radiation, 10μl MTT reagent was added into each well and incubated in a humidified 37 ºC atmosphere for another 4 hs. Then optical density values were measured by Bio-Rad model 680 Microplate Reader at a wavelength of 570 nm.

For colony formation assay, cells were seeded into the 12 well culture plates with 3x10^3^-5x10^3^ /well. In the next day, cells were treated with radiation and then continue incubation for up to 2 weeks. Then the plates were stained with crystal violet, and the colony count was evaluated with the Image J software.

*Western blotting and Co-immunoprecipitation (Co-IP).* Western blotting was performed to detect the specific protein levels (total or phosphorylated). Cells were lysed in RIPA buffer supplemented with PhoStop (Roche, Swiss) on ice. After BCA protein quantification, loading buffer was added into the mixture. Then standard Western Blotting procedure was applied. Nitrocellulose filter membrane with protein was covered with primary antibodies overnight. After TBST washed the membrane three times, HRP (horse radish peroxidase)-conjugated anti-mouse, anti-goat or anti-rabbit antibodies were applied according to the primary antibodies the next day. After TBST washed the membrane three times, ECL (electrochemiluminescence) showed protein levels.

For Co-IP, cells after radiation were lysed by RIPA solution for Co-IP on ice for 30 mins. Then BCA protein quantification was applied. Each adjusted buffer was added into 5 μl anti-β-arrestin1antibody (Santa Crus) and protein A and G sepharose beads (Invitrogen) overnight. After washing three times with an immunocomplex wash buffer, 1X sample buffer was added into the immunoprecipitates and the mixture was boiled for 10 mins. Then western blotting was applied.

*Flow Cytometry.* PE-Annexin V/7-Amino-ActinomycinD (7-AAD) staining assay was used to test apoptosis. Annexin V is a phospholipid-binding protein with a high affinity for PS. In normal live cells, PS is located on the cytoplasmic surface of the cell membrane. When cells undergo apoptosis, PS is translocated from the inner to the outer leaflet of the plasma membrane, and becomes available for Annexin V binding. 7-AAD is generally cell-impermeable for live cells, and undergoes a spectral shift upon association with DNA, so it has been used to label necrotic or late apoptotic/dead cells with damaged cell membranes. Therefore, combination of PE-Annexin V and 7-AAD stains can be exploited to distinguish cells undergo necrosis and apoptosis to cell death. The apoptosis was detected in this assay with flow cytometer (FCM). PE Annexin V Apoptosis Detection kit (BD Pharmingen, USA) and LSRFortessa (BD Pharmingen, USA) were used in flow cytometry. In brief, cells were harvested by trypsin 10 hs after radiation treatment. Then cells were washed three times with cold PBS and centrifuged at 1000 rpm for 5 mins each time. Samples were added into binding buffer and suspended with a density of 10^6^/ml. Then the mixture were sustained with the PE-Annexin V/7-AAD for 10 mins and analyzed with flow cytometry.

*COMET assay.* Comet assay was performed to evaluate the DNA damage induced by radiation according to the manufacturer's instructions (Cell Biolabs, USA). Cells were digested by trypsin and washed twice with cold PBS (2x10^5^ /ml). Then OxiSelect COMET Slides were covered by the mixture of COMET agarose and cells. After lysis, the slides underwent electrophoresis for 30 mins under 1 volt/cm. Cells were stained with Vista Green DNA dye and visualized by the epifluorescence microscopy. Tail length, head length, and tail moment were analyzed by COMET-Assay IV software V4.3 (Perceptive Instruments, UK).

*Calcein AM/Propidium Iodide staining and spectroscopic analysis.* The Calcein AM is a non-fluorescent dye that can enter into live cells. Once inside, the AM (acetomethoxy) is cleaved by esterases and the fluorescent calcein protein remains inside the cells. Although Propidium Iodide (PI) does not pass through the living cell membrane, it can pass through the damaged cell membrane and stain the nucleus. And we used this assay to evaluate cell viability after radiation with the manipulation of β-arrestin1 status in H520 cell line. The cell seeding, treatment durations, and radiation protocol were similar to previously described assays. After 10hs of 2Gy radiation, we aspirated the cell culture medium and incubated the cells with 100 μl medium containing 2 μM Calcein AM and 4 μM PI for 15 mins at 37 °C in 5% CO_2_. We calculated cell viability by measuring the absorbance at 490±10/545 nm and comparing with the control cells.

*Animal model and radiation procedure.* All mouse experimental procedures were performed in accordance with the Regulations for the Administration of Affairs Concerning Experimental Animals approved by the State Council of People's Republic of China. And the study was approved by the Hospital Ethical Committee. All methods used in this study were carried out in accordance with the approved guidelines.

BALB/c-nu mice (Female, 5-6 weeks old) were purchased from Vital River Laboratory Animal Technology (VRL, China). Mice were bred and maintained under specific-pathogen-free conditions. H1299 cells (1x10^6^) were injected subcutaneously in the left flank of mouse. Tumor growth was monitored and measured in volume (length x width x width/2) at the indicated time points during a 28-day period after tumor volume reaches 100mm^3^. Mice were divided into 4 groups with matched weight: 1 the group treated with radiation after in vivo transfection of vector (Vector group); 2 the group treated with radiation after in vivo transfection of ARRB1 (ARRB1+ group);^3^ the group treated with radiation after in vivo transfection of control siRNA(Control siRNA group); 4 the group treated with radiation after in vivo transfection of ARRB1 siRNA(ARRB1- group). Each group consisted of 5 animals. ARRB1 Plasmid (1.5 mg/kg) was mixed with transfection reagent (Entranster- in vivo, Engreen), and then was intratumor injected in ARRB1+ group every two days. ARRB1 siRNA (5 mg/kg) was mixed with transfection reagent (Entranster- in vivo, Engreen), and then was intratumor injected in ARRB1- group every two days. Each animal was anaesthetised by intraperitoneal injection of 0.2 ml 0.3 % pentobarbital sodium(50 mg/kg). The anaesthetised animal was laid face-up and fixed on a platform with Primus 3831 linear accelerator (Siemenscompany, Germany). A 1 cm^2^ (1 cmx1 cm) area of the lower abdomen of the animal was chosen and marked for the radiation. A set of lead shields were laid upon the animal in a manner that allowed only the marked area to remain uncovered. All of the mice received a 5-Gy dose of 300 keV X-rays at a dose rate of 3.6 Gy min^-1^ once a week. After 4 weeks, animals were euthanized^17^.

*Statistical analysis.* All statistical calculations were performed by SPSS 20.0 for Windows (SPSS Inc, USA) and Graphpad prism 6 (GraphPad Software, USA). Each test was performed triplicate independent times. And data were expressed in the form of count or mean ± standard deviation and analyzed by t or χ^2^ test according to the data character (Continuous variables: t test; Categorical variables: χ^2^ test). For all calculations, p values were all two-sided and less than 0.05 were significant.

## Results

**Figure 1:**
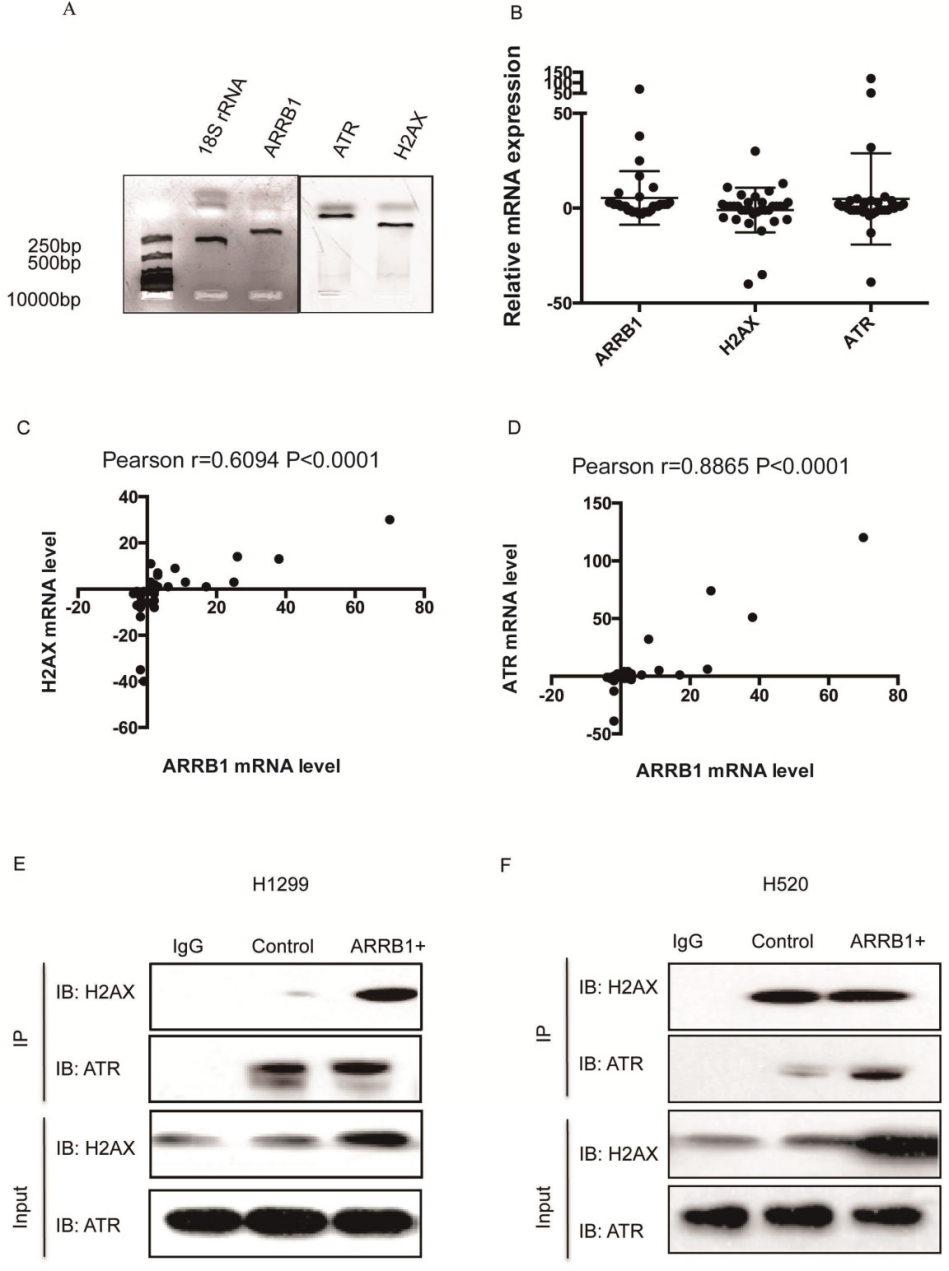
** β-arrestin1 mRNA level co-relates with ATR and H2AX, and interacts with ATR and H2AX.** A. All fragments amplified were around or less than 250 bp, and were suitable for the qRT-PCR with high specificity. B. The fold change of expression between carcinoma and para-carcinoma was assessed by 2^-ΔΔ^ Cycle threshold (Ct) value. C. There was moderate co-relation between ATR and β-arrestin1 mRNA level (Pearson r=0.6094, p<0.0001). D There was high co-relation bwtween H2AX and β-arrestin1 (Pearson r=0.8865, p<0.0001). E and F, β-arrestin1 bound to both ATR and H2AX, and this binding could be enhanced by radiation in H520 and H1299 cell lines.

*Β-arrestin1 mRNA level co-relates with ATR and H2AX and β-arrestin 1 interacts with ATR and H2AX.* To explore the potential relation between β-arrestin1, H2AX and ATR, we used quantitative real-time polymerase chain reaction (qRT-PCR) technic to investigate the mRNA level in 40 pairs of lung carcinoma and para-carcinoma tissues from Bio-bank, Shandong Provincial Hospital. All primers were amplified by polymerase chain reaction and tested by agarose gel electrophoresis (Fig 1A). All fragments amplified were around or less than 250 bp, and were suitable for the qRT-PCR with high specificity. The difference of expression between carcinoma and para-carcinoma was assessed by 2^-ΔΔ^ Cycle threshold (Ct) value (ΔCt=Ct(ATR, β-arrestin1 or H2AX)-Ct(18SrRNA), ΔΔCt=ΔCt(carcinoma)-ΔCt(para-carcinoma), Fig 1B). However, there was moderate to high co-relation between ATR and β-arrestin1 mRNA level (Pearson r=0.6094, p<0.0001, Fig 1C) as well as H2AX and β-arrestin1 (Pearson r=0.8865, p<0.0001, Fig 1D). The potential association between β-arrestin1with ATR or H2AX indicated that β-arrestin1 might bind to ATR and H2AX directly or indirectly. That binding was proved by Co-IP test. As shown in Fig 1E and 1F, β-arrestin1 bound to both ATR and H2AX, and this binding could be enhanced by radiation in H520 and H1299 cell lines.

**Figure 2:**
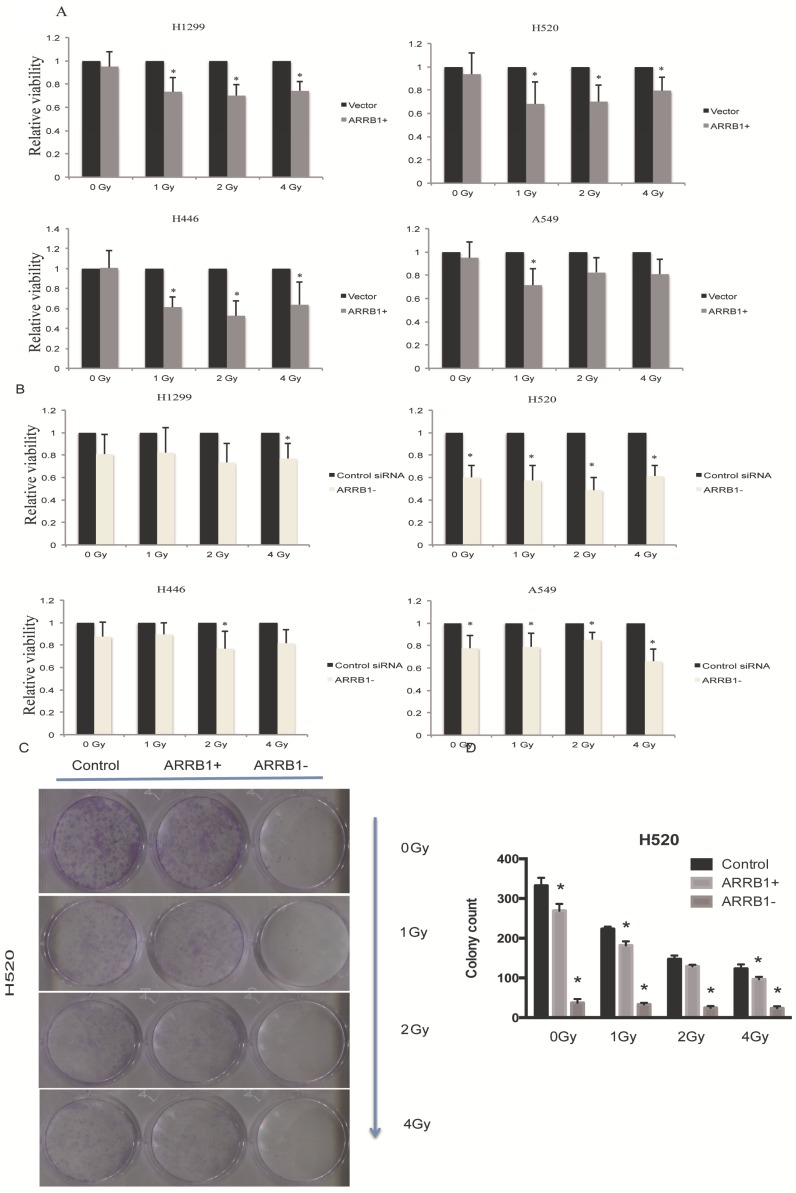
** Manipulation of β-arrestin1 status inhibits lung cancer cell growth. A.** β-arrestin1 could inhibit cell growth at different dosage of radiation in 4 NSCLC cell lines, especially at 1 and 2 Gy. B. Knockdown of β-arrestin1 could lead to proliferation suppression. C. The colony formation assay results were in accordance with MTT results.

*Manipulation of β-arrestin1 status inhibits lung cancer cell growth.* Our results showed that overexpression of β-arrestin1 could suppress cell viability at different dosage of radiation in all 4 NSCLC cell lines, especially at 1 and 2 Gy dosage (Fig 2A). And what interested us most is that knockdown of β-arrestin1 could lead to proliferation suppression as well (Fig 2B). The colony formation assay results were in accordance with MTT results (Fig 2C), which was that manipulation of β-arrestin1 status reduced lung cancer cell growth. We conducted Calcein AM/Propidium Iodide staining assay to validate our results of the impact of β-arrestin1 on cell viability with radiation in Supplementary Figure 1. Our result showed that overexpression and knockdown of β-arrestin1 could both suppress cell viability.

**Figure 3:**
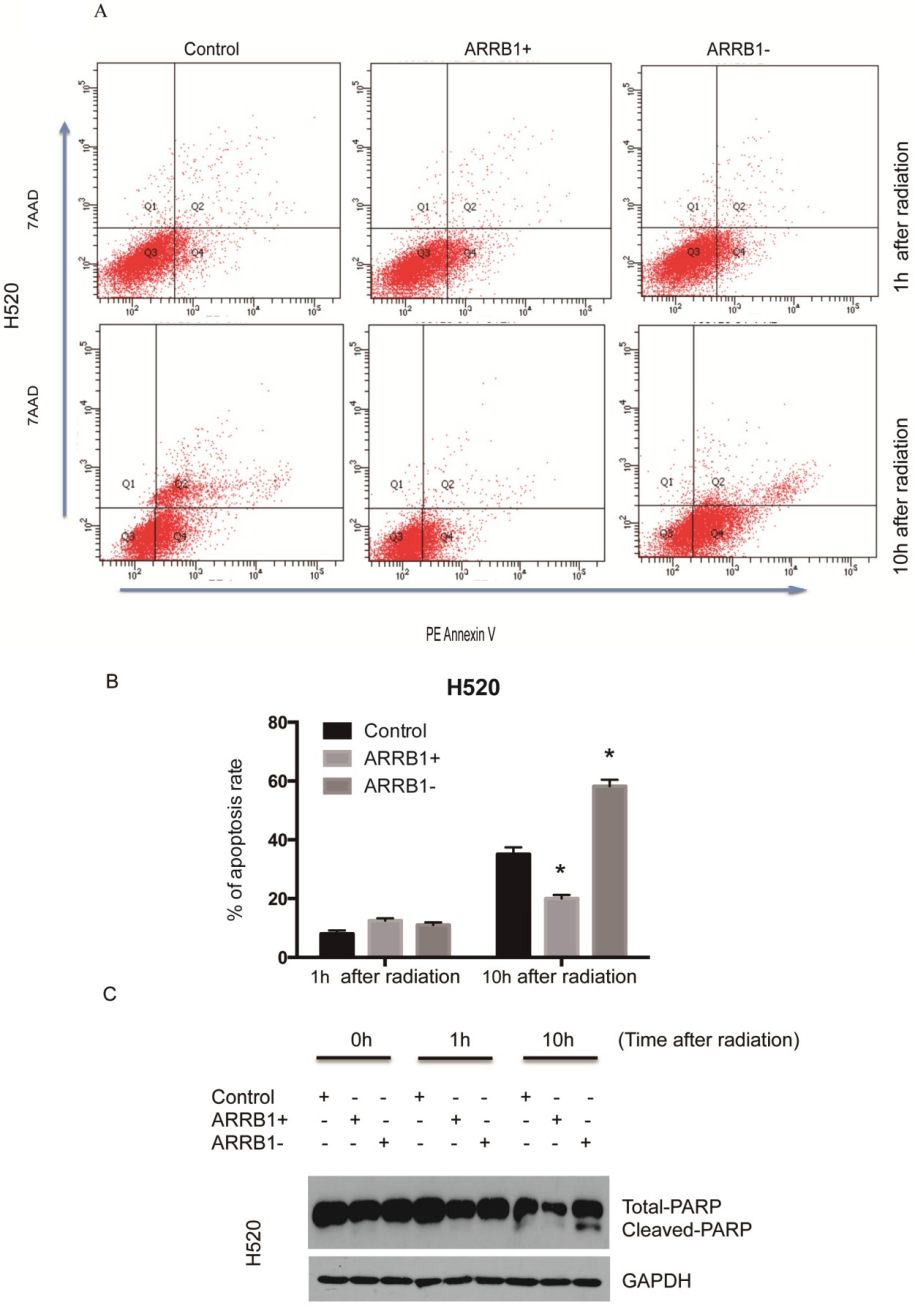
** β-arrestin1 status regulates radiation-induced apoptosis.** A. Knockdown of β-arrestin1 enhanced apoptosis significantly at 10 hs after 2 Gy radiation. B. Overexpression of β-arrestin1 reduced apoptosis. C. Cleaved PARP showed up at 10 hs after 2 Gy radiation, especially at β-arrestin1 knockdown lane.

**Figure 4:**
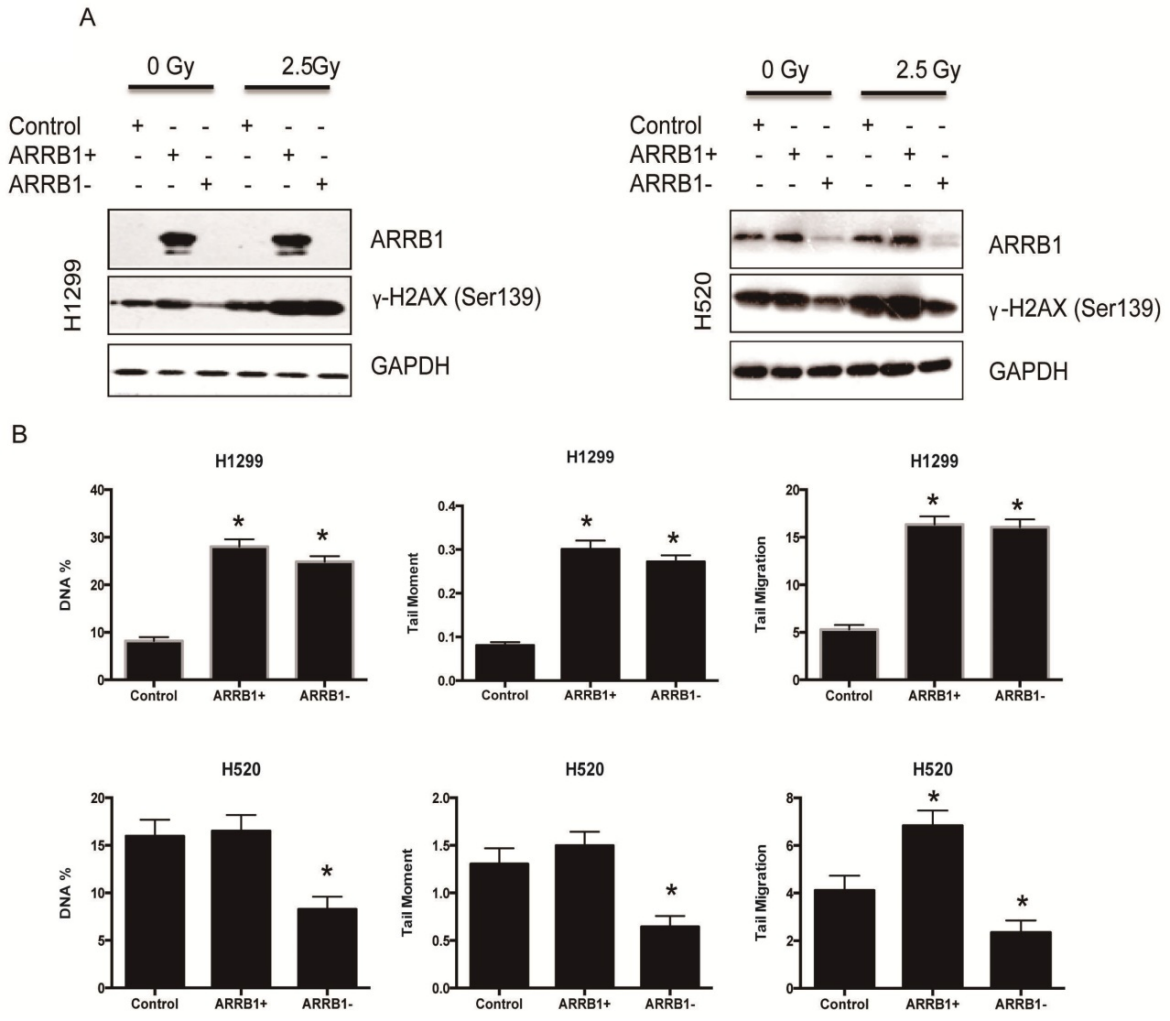
** β-arrestin1 status regulates radiation-induced DNA damage.** A. The phosphorylation level of H2AX was parallel to β-arrestin1 expression with 2.5 Gy radiation treatment. B. Comet assay results showed that DNA damage was enhanced with β-arrestin1 overexpressed in H1299 cell line, and it was inhibited with β-arrestin1 knockdown in H520 cell line.

*Β-arrestin1 status regulates radiation-induced apoptosis and DNA damage.* Flow cytometry was used to test the apoptosis rate after manipulation of β-arrestin1 status, and our results showed that knockdown of β-arrestin1 enhanced apoptosis significantly at 10 hs after 2 Gy radiation, in the other hand, overexpression of β-arrestin1 enhanced apoptosis slightly at 1 h after 2 Gy radiation and inhibited apoptosis significantly at 10 hs (Fig 3A and B). Western blotting results indicated that cleaved PARP showed up at 10 hs after 2 Gy radiation, especially at β-arrestin1 knockdown lane (Fig 3C). The phosphorylation level of H2AX was parallel to β-arrestin1 expression with 2.5 Gy radiation treatment (Fig 4A). Comet assay results showed that DNA damage was enhanced with β-arrestin1 overexpressed in H1299 cell line, and it was inhibited with β-arrestin1 knockdown in H520 cell line (Fig 4B).

**Figure 5:**
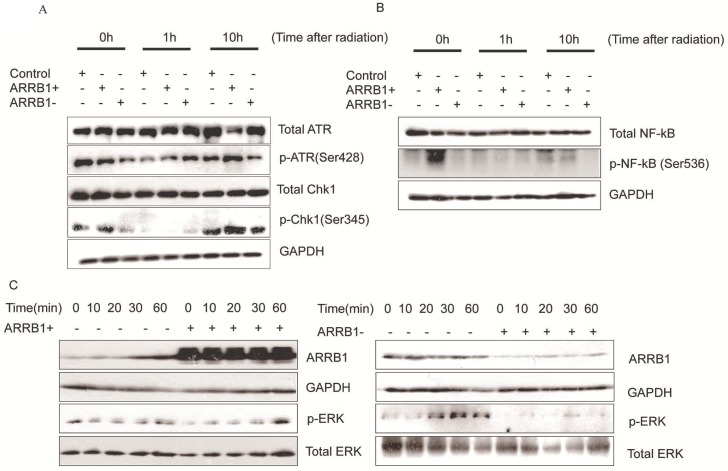
** β-arrestin1 mediates radiation-induced signaling pathway.** A. Phosphorylation of ATR and Chk1 were parallel to β-arrestin1 status, especially at 0 and 10 hs after radiation. B,C,D. radiation could activated ERK and NF-kB pathways within 60 minutes.

*Β-arrestin1 mediates radiation-induced signaling pathway.* To investigate the impact of β-arrestin1 on DDR pathway, we test the protein expression within a certain period of time after radiation. Our results showed that phosphorylation of ATR and Chk1 were parallel to β-arrestin1 status, especially at 0 and 10 hs after radiation (Fig 5A). To investigate the effect of radiation on cellular signaling pathways, we tested the protein expression at time points of 0, 10, 20, 30, 60 mins after radiation. Our results showed that radiation could activated ERK and NF-kB pathways within 60 mins (Fig 5 B, C, D).

**Figure 6:**
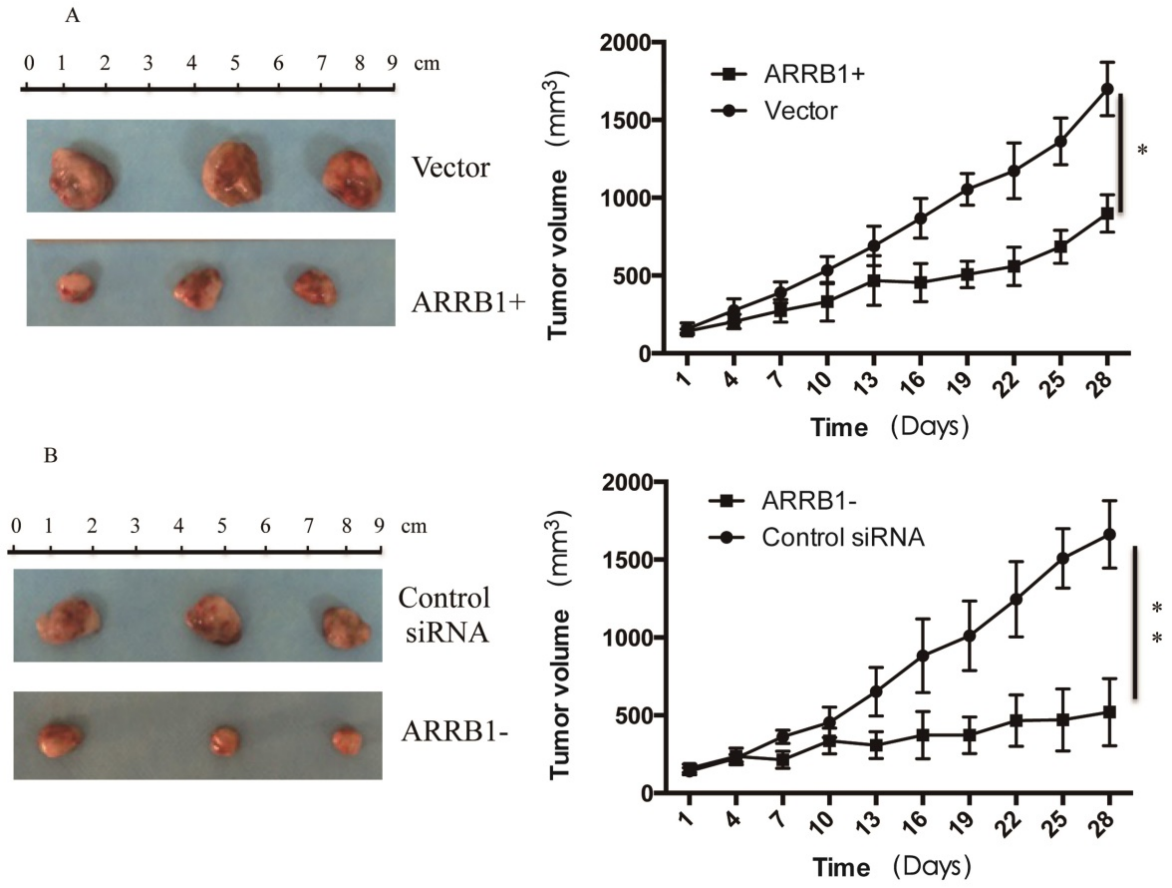
** Manipulation of β-arrestin1 status regulate the response to radiation in vivo.** Tumor growth was delayed with overexpression or knockdown of β-arrestin1, and manipulation of β-arrestin1 status could enhance radiation sensitivity in vivo.

*Manipulation of β-arrestin1 status regulate the response to radiation in vivo.* To analyze the regulation effect of β-arrestin1 on radiation in vivo, we established mouse xenograft models and analyzed the effect of β-arrestin1 on tumor growth. Mice were divided into 4 groups with matched weight: 1 the group treated with radiation after in vivo transfection of vector (Vector group, n=5); 2 the group treated with radiation after in vivo transfection of ARRB1 (ARRB1+ group, n=5); 3 the group treated with radiation after in vivo transfection of control siRNA(Control siRNA group, n=5); 4 the group treated with radiation after in vivo transfection of ARRB1 siRNA(ARRB1- group, n=5). ARRB1 Plasmid (1.5 mg/kg) was mixed with transfection reagent (Entranster- in vivo, Engreen), and then was intratumor injected in ARRB1+ group every two days. All of the mice received a 5-Gy dose of 300 keV X-rays at a dose rate of 3.6 Gy min^-1^ once a week. Tumor growth was delayed with overexpression or knockdown of β-arrestin1, and manipulation of β-arrestin1 status could enhance radiation sensitivity in vivo (Fig 6).

## Discussion

In our study, we investigated the impact and potentially relevant mechanism of β-arrestin1 on radiotherapy sensitivity. Our data showed that β-arrestin1might take part in alteration of radiotherapy sensitivity mainly through two mechanisms: 1) enhancement of DNA damage and apoptosis; 2) regulation of cell proliferation. Overexpression of β-arrestin1 enhances DNA damage inducing apoptosis in the early stage. Meanwhile, depletion of β-arrestin1 inhibits cell proliferation through the suppression of cell pathways including NF-kB and ERK.

When DNA damage occurs, cell promptly monitors this heritable error and evokes cell cycle arrest and then chromosome repair. If the damage was so severe that repair could not be completed, it turns to apoptosis 18. It was reported that ATM could interact with downstream p53 and induce apoptosis in response to DNA damage 19. However, this mechanism might not be predominant in acute radiation 20. Radiation inducing apoptosis was reported to be caspase-dependent 21. Radiation could cause cytochrome C release from mitochondria into cytosol, which can be blocked by Bcl-2 22. Cytoplasmic cytochrome C caused downstream caspases cascade activation 23. Downstream caspase 9 interacted with Apaf-1 and activated caspase 3,6,7 complex 24. Caspase 3 was a key component of caspase family and apoptosis cascade. Under radiation, caspase 3 could trigger breakdown of BID at Asp-59 and activate BID 25, 26. In return, tBID promote the upstream release of mitochondrial cytochrome C to cytosol via Bak thus forming a positive feedback 27. Therein, cleavaged β-arrestin1 enhanced the effect of tBID on release of cytochrome C and promoted apoptosis 14.

As a pathway, DDR also has sensors, transducers and effectors. PARP (poly ADP-ribose polymerase) was found to be one of the first steps of DDR and provoked downstream of this pathway 28. Then a class of phospho-inositide kinase (PIK)-related proteins was activated as one of the transducers. Therein, ATM and ATR were reported as the main upstream DDR kinases 29. Downstream of these two kinases were two families of checkpoint kinases (Chk1 and Chk2), BRCA1, p53 and so on 30. Ultimately, through this pathway, cell cycle arrest, DNA repair and transcription regulation occurred. Our results indicated that after radiation, transfection of β-arrestin1plasmids could enhance DDR pathway activation including ATR and downstream Chk1, BRCA1 phosphorylation, thus more γ-H2AX was tested. That indicated that elevated β-arrestin1 also enhance more DNA repair through binding to ATR and γ-H2AX, which could patricianly explain that reduced apoptosis was detected at 10 hs after radiation (Fig 3).

Our results showed that radiation could phosphorylate NF-kB and activate NF-kB pathway. Stilmann reported that PARP-1 could assemble IKKγ, and then promote IKK and NF-kB activation as well as NF-kB induced resistance to apoptosis 31. The activation of NF-kB pathway was reported to be associated with radio-resistance and anti-apoptosis, and β-arrestin1 participated in the activation of NF-kB pathway 32-36. Apart from NF-kB pathway, constitutive activation of PI3K/AKT and ERK pathways was also reported to participate in radio-resistance 37, 38. Specifically, MAPK/ERK pathway took part in DNA repair, anti-apoptosis, NF-kB pathway activation and thus induced radio-resistance 39, 40. In our study, transfection with β-arrestin1 could enhance ERK activation and knockdown with siRNA could suppress this pathway. Thus knockdown β-arrestin1 could enhance the radio-sensitivity via suppression of cell proliferation, cell survival and anti-apoptosis induced by ERK, NF-kB pathways.

In the clinical study of exploring the effect of β-arrestin1on breast cancer treatment and Tamoxifen response, high and absent stromal expression β-arrestin1 were both related to a poor outcome 41. That evidence indicated that β-arrestin1functioned diversely under different circumstances. To sum up, β-arrestin1 plays different roles in radiotherapy, and manipulating of β-arrestin1 could increase radio-sensitivity for lung cancer. Our study may need further study and the researchers to try out up- or down-expression of β-arrestin1in radiotherapy in the clinical application.

## Supplementary Material

Supplementary figures and tables.
