# Supplementary material for: Regulation of response to radiotherapy by β-arrestin1 in Non-small cell lung cancer
Source: J Cancer. 2019 Jul 8;10(17):4085–95. doi: 10.7150/jca.30012 (PMC6692618; doi:10.7150/jca.30012)
Supplement: Supplementary file 1 — Supplementary figures and tables. [file jcav10p4085s1.pdf]

Supplementary Fig 1. A

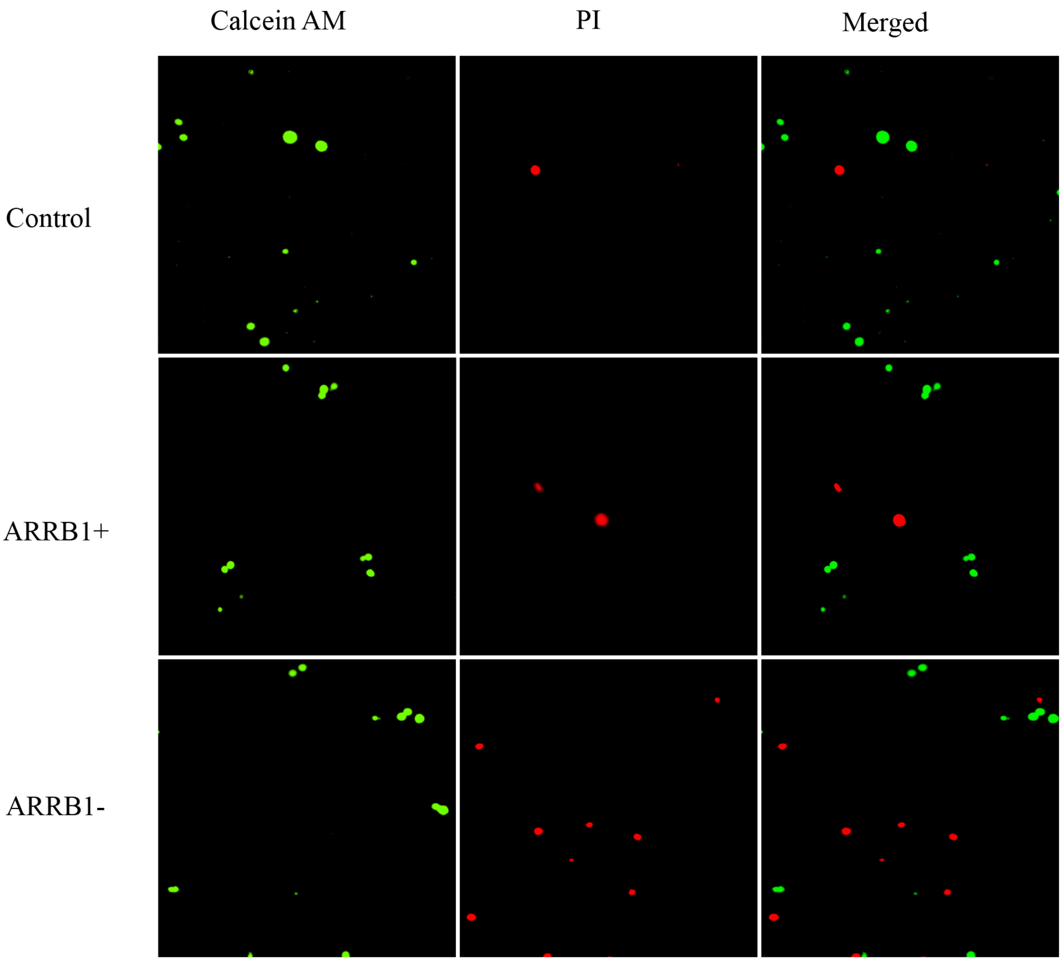

B

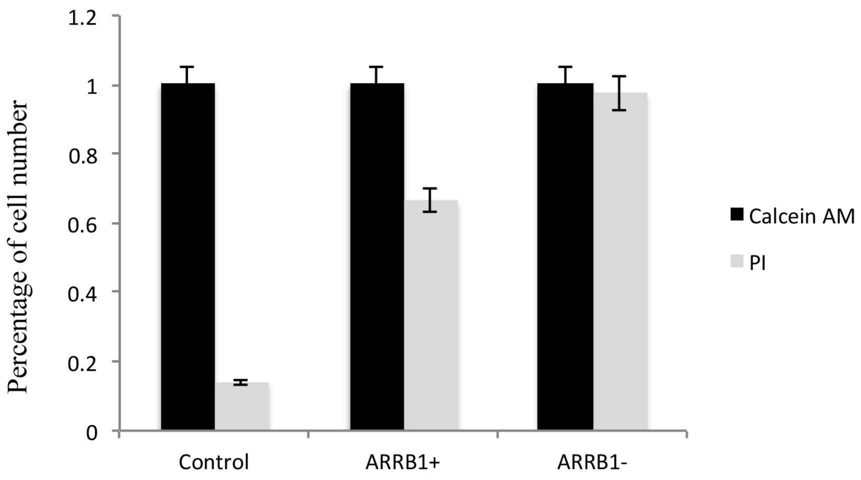

Supplementary Figure 1. Calcein AM/PI assay to evaluate cell viability after 2 Gy radiation treatment with the manipulation of β-arrestin1 status in H520 cell line. Fluorescent microscopic images and quantitative graphs show the live and dead cell populations.

Variables	Number(%)
Gender	
Male	28(70.0)
Female	12(30.0)
Age	
<60	19(47.5)
≥60	21(52.5)
Differentiation	
Well/moderate differentiation	24(60.0)
Poor differentiation	16(40.0)
Histology	
Adenocarcinoma	23(57.5)
Squamous cell carcinoma	16(40.0)
Other	1(2.5)
Venous invasion	
Negative	5(12.5)
Positive	35(87.%)
TNM staging	
I	17(42.5)
II	16(40.0)
III	7(17.5)

Supplementary table 1. Patients and tumor characteristics.
